# Biomarker discovery for colon cancer using a 761 gene RT-PCR assay

**DOI:** 10.1186/1471-2164-8-279

**Published:** 2007-08-15

**Authors:** Kim M Clark-Langone, Jenny Y Wu, Chithra Sangli, Angela Chen, James L Snable, Anhthu Nguyen, James R Hackett, Joffre Baker, Greg Yothers, Chungyeul Kim, Maureen T Cronin

**Affiliations:** 1 Genomic Health, Inc. Redwood City, CA, USA; 2 National Surgical Adjuvant Breast and Bowel Project (NSABP), Pittsburgh, PA, USA

## Abstract

**Background:**

Reverse transcription PCR (RT-PCR) is widely recognized to be the gold standard method for quantifying gene expression. Studies using RT-PCR technology as a discovery tool have historically been limited to relatively small gene sets compared to other gene expression platforms such as microarrays. We have recently shown that TaqMan^® ^RT-PCR can be scaled up to profile expression for 192 genes in fixed paraffin-embedded (FPE) clinical study tumor specimens. This technology has also been used to develop and commercialize a widely used clinical test for breast cancer prognosis and prediction, the Onco *type*DX™ assay. A similar need exists in colon cancer for a test that provides information on the likelihood of disease recurrence in colon cancer (prognosis) and the likelihood of tumor response to standard chemotherapy regimens (prediction). We have now scaled our RT-PCR assay to efficiently screen 761 biomarkers across hundreds of patient samples and applied this process to biomarker discovery in colon cancer. This screening strategy remains attractive due to the inherent advantages of maintaining platform consistency from discovery through clinical application.

**Results:**

RNA was extracted from formalin fixed paraffin embedded (FPE) tissue, as old as 28 years, from 354 patients enrolled in NSABP C-01 and C-02 colon cancer studies. Multiplexed reverse transcription reactions were performed using a gene specific primer pool containing 761 unique primers. PCR was performed as independent TaqMan^® ^reactions for each candidate gene. Hierarchal clustering demonstrates that genes expected to co-express form obvious, distinct and in certain cases very tightly correlated clusters, validating the reliability of this technical approach to biomarker discovery.

**Conclusion:**

We have developed a high throughput, quantitatively precise multi-analyte gene expression platform for biomarker discovery that approaches low density DNA arrays in numbers of genes analyzed while maintaining the high specificity, sensitivity and reproducibility that are characteristics of RT-PCR. Biomarkers discovered using this approach can be transferred to a clinical reference laboratory setting without having to re-validate the assay on a second technology platform.

## Background

Over the last decade, many studies have applied gene expression analysis to identify biomarkers for prognostic and/or predictive information in relation to human disease [1-4]. RNA for these studies has come from either frozen or formalin fixed paraffin embedded (FPE) tissue. RNA from frozen tissues is generally regarded as the most desirable for molecular assays, since if collected correctly it is generally intact and can be analyzed by a wide variety of standard molecular biology techniques. However, FPE tissue is the most widely available source of tumor tissue as it is the product of standard tissue processing procedures followed by surgical pathology laboratories. It is clear that RNA obtained from FPE tissue is not full length and the extent of degradation increases with storage time [5]. Therefore, it is generally considered to be challenging to extract RNA from archival FPE tissue for analysis by standard molecular biology techniques.

With the development of automated liquid handling and DNA microarrays, high throughput screening using hundreds of samples and hundreds or thousands of genes has become routine in many laboratories. DNA microarrays offer the advantage of simultaneously assessing the relative expression level of thousands of genes with a relatively small amount of starting RNA. However, DNA microarray measurements are limited in dynamic range, specificity and reproducibility, leading to high false positive and false negative biomarker discovery rates. As currently configured, DNA microarray technology also requires high quality RNA. Alternatively, reverse-transcription polymerase chain reaction (RT-PCR) technology offers the advantages of high accuracy and reproducibility, and precise quantitation over a wide dynamic range. To overcome the issue of fragmented RNA in FPE tissue specimens, assays can be optimized for short amplicons so the RNA from FPE tissue can be successfully analyzed [5].

It has been suggested that there is a bottleneck in scaling up TaqMan^® ^RT-PCR using archival FPE samples to analyze beyond 30 genes [6]. On the contrary, we demonstrated that TaqMan^® ^RT-PCR biomarker screening using FPE samples is not necessarily limited to small candidate gene sets and have performed studies with up to 192 genes [5,7,8]. Using this technology we developed and commercialized a 21 gene panel that predicts the likelihood of cancer recurrence in early stage breast cancer patients [9]. We have now scaled our TaqMan^® ^RT-PCR screening process to assay 768 wells of data per patient sample. Here we describe the methodology that was used to identify prognostic biomarkers in stage II/III colon cancer [10].

In this paper we report the use of this high throughput, highly parallel TaqMan^® ^RT-PCR process to screen RNA extracted from colon cancer FPE clinical trial specimens (NSABP C01 and C02 studies). We focused on selecting candidate genes known to be involved in pathways related to colon cancer and genes from published expression profiling data sets relating to colon cancer prognosis and response to therapy. Our results indicate that this approach yields high quality expression data that can be used for simultaneous evaluation of hundreds of candidate genes in defined cohorts of patients to identify prognostic and predictive colon cancer biomarkers.

## Results

### Large scale multiplexed, gene-specific reverse transcription

**Figure 1:**
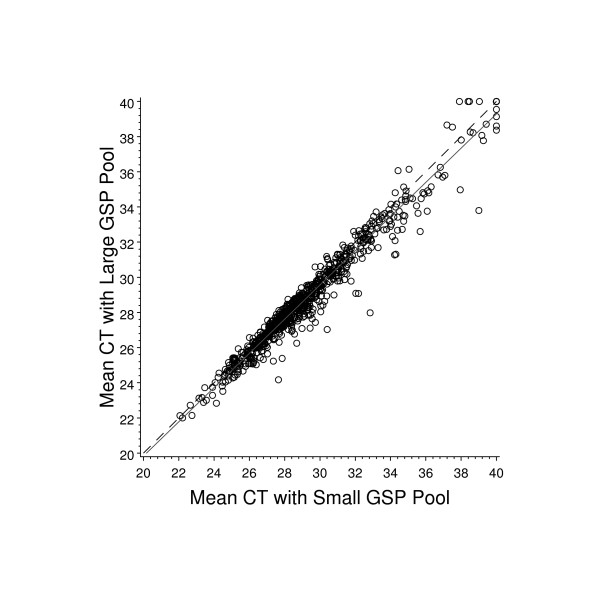
**Effect of increasing priming complexity in reverse transcription using high quality RNA template**. Eight gene specific primer (GSP) pools each containing from 94 to 96 unique primers were used to prime separate RT-PCR reactions with high quality commercial RNA template. These 8 GSP pools were then combined to make a single GSP pool that was used to prime one RT-PCR reaction using the same template RNA. Both priming methods were performed twice and the average C_T _value for each gene was determined for this analysis. The solid line represents the least squares line fit and the dashed line represents the line of concordance.

FPE RNA is largely fragmented, making it a poor substrate for poly-dT primed reverse transcription. Random priming in combination with poly-dT priming has proven to be somewhat more successful for generating cDNA from FPE RNA [6,11]. However, with highly fragmented RNA, even random priming does not yield satisfactory RT-PCR data [see Additional file 1], as compared to that obtained with in tact RNA [see Additional file 2]. We therefore chose to maximize assay sensitivity by using gene specific priming for each target sequence included in the RT-PCR screening panel. To evaluate the quality and consistency of cDNA from this complex reverse transcription reaction, we compared the expression values resulting from 8 smaller reverse primer pools (each containing 94 to 96 primers) to those from the single pool with all of the primers combined. In every case, cDNA from multiplexed RT reactions was combined with PCR master mix and distributed into 384 well plates with each well containing the primers and probe for 1 gene assay. High quality commercial RNA (Universal RNA, Stratagene, La Jolla, CA.) was used as the template for this evaluation. The resulting data are shown in Figure 1. Each priming condition was repeated twice and the mean C_T _values were plotted. The mean C_T_values were very similar: C_T _= 29.40 versus C_T _= 28.98 for the small and large gene-specific primer (GSP) pools, respectively. Additionally, the Pearson's correlation for these two priming methods was 0.98. The median difference between priming methods was 0.4 C_T _units. The large majority of assays (97.5%) had a C_T _difference less than 2. For 2 of the assays which had a C_T _difference greater than 2, this could be accounted for by 1 failed reaction (C_T _= 40). The priming efficiency for all other assays where alternative priming lead to a C_T _difference greater than 2 appears to be reproducible so the result may be accounted for by cross-priming events in the more complex GSP pool.

**Figure 2:**
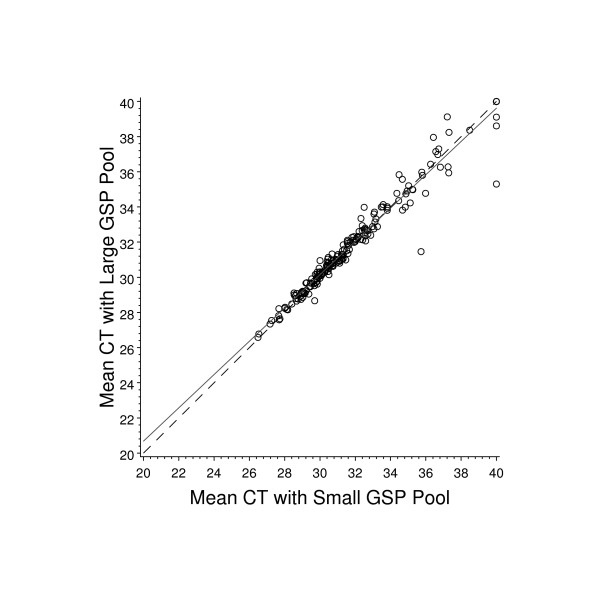
**Effect of increasing priming complexity reverse transcription using FPE tissue RNA template**. Eight GSP pools containing from 94 to 96 unique primers were prepared. Two pools were selected at random to prime separate RT-PCR reactions using RNA from FPE tissue. The 8 GSP pools were then combined to make a single GSP pool that was used to prime one RT-PCR reaction using the same FPE RNA template. The data therefore represents concordance between primings for the subset of gene assays represented with those two GSP pools. Both priming methods were performed twice and the average C_T _values determined for this analysis. The solid line represents the least squares line fit and the dashed line represents the line of concordance.

While measuring the realistic limit of multiplexed RT-priming in FPE RNA we did not want the data complicated by biological variability contributed by individual samples. We therefore created a sample consisting of pooled FPE RNA that represented a range of high and low expression values across the gene panel and reflected the quality of samples that would be used in the gene identification study. This was done by pooling 36 breast FPE RNA samples from tumor blocks over 10 years old. Two of the 8 primer sub-pools were selected at random to verify that the priming reaction remained consistent as the RNA template type changed from a high quality sample to a highly fragmented sample. The results, shown in Figure 2, indicate that the reaction using FPE RNA as a template was consistent with that seen for the high quality commercial RNA. The Pearson's correlation was 0.95 and the mean C_T _values obtained for the entire gene panel were almost identical: C_T _= 31.84 versus C_T _= 31.79 using the small and large gene-specific primer (GSP) pools, respectively. The average raw C_T _obtained with the FPE RNA is higher than that obtained with the high quality RNA. This is expected since the FPE RNA is highly fragmented resulting in fewer available targets for priming [5]. Although this shift does result in FPE samples being somewhat skewed toward the upper end of the assay dynamic range, typically at least a 2000 fold range in expression can still be reliably measured for most genes before reaching the assay limit of quantitation [12].

Once we confirmed the high complexity priming reaction was working consistently in FPET RNA, we prepared a gene specific primer pool containing 761 unique reverse primers for the biomarker discovery study using NSABP C01 and C02 clinical trial specimens.

### Sample exclusion

To be included in the final study analysis, samples had to pass pathology, clinical and laboratory data QC requirements. To meet pathology acceptance criteria, a minimum of 5% of the tissue present in each sample was required to be invasive cancer cells. All samples were dissected to enrich tumor tissue and minimize non-tumor elements.

RNA was extracted from 354 FPE tissues cut from the NSABP C01/C02 clinical study samples (archived from 1977–1983) [13,14]. All samples yielded RNA using the semi-automated extraction process described below, however a total of 38 samples yielded less than the 1069 ng RNA required to ensure a final concentration of 1 ng/assay well. We performed RT-PCR on 23 of these samples, but none of these yielded acceptable RT-PCR data. Of the 306 samples taken through RT-PCR with the standard RNA load, 21 (< 7%) were excluded from analysis due to unacceptable RT-PCR data. Although the precise age of each excluded block was not available, it is reasonable to assume that the samples with unacceptable RT-PCR data may have been from the oldest blocks [5]. After other pre-specified exclusion criteria were applied, (pathological and clinical ineligibility), the final number of evaluable patients was 270. Table 1 provides a detailed breakdown of all excluded samples. It should be noted that the overall exclusion rate in this study is higher than we have experienced in other studies and is very likely related to the age of these samples, which is the oldest set we have analyzed.

**Table 1:** Sample exclusion criteria

	Number excluded	Percent excluded	Remaining samples
Samples extracted	N/A	0	354
Insufficient RNA	-38	10.7%	316
Incomplete sample data	-4	1.1%	312
Unsatisfactory qPCR	-21	5.9%	291
Pathologically ineligble	-10	2.8%	281
Clinically ineligble	-11	3.1%	270
**Total in final dataset**		76.3%	270

Samples were excluded on the basis of several different categories. Various acceptance criteria were applied to the dataset. The table shows how many samples were excluded from the final dataset based on each category. There were 354 samples extracted and the final evaluable data set was 270.

### Process throughput

Using a semi-automated extraction process, two lab technicians extracted RNA from 48 samples per day. Paraffin was removed from the tissue manually with xylene, followed by ethanol washes. After a proteinase K digestion and phenol-chloroform purification, the remaining RNA extraction steps were performed on an automated liquid handler using a plate-based protocol. The purified RNA was then quantified using an automated RiboGreen fluorescence assay (Invitrogen, Carlsbad, CA.). The resulting files containing the RNA quantification data were automatically collected by the laboratory information management system (LIMS). The LIMS used those data to generate an RNA concentration normalization work-list, which was then executed by an automated liquid handler during the reverse transcription reaction assembly procedure. The reverse transcription reaction was completed using an MJ Research thermocycler (Bio-Rad, Hercules, CA). Finally, quantitative PCR reactions were assembled at a rate of 64 plates (32 patient samples) per day. All automated liquid handlers were obtained from Tecan (TECAN Schweiz AG, Männedorf, Switzerland). This system yielded a total of 24,576 real time quantitative PCR reactions to be performed per day using four ABI PRISM 7900 HT instruments (Applied Biosystems, Foster City, CA.).

### Process reproducibility

**Figure 3:**
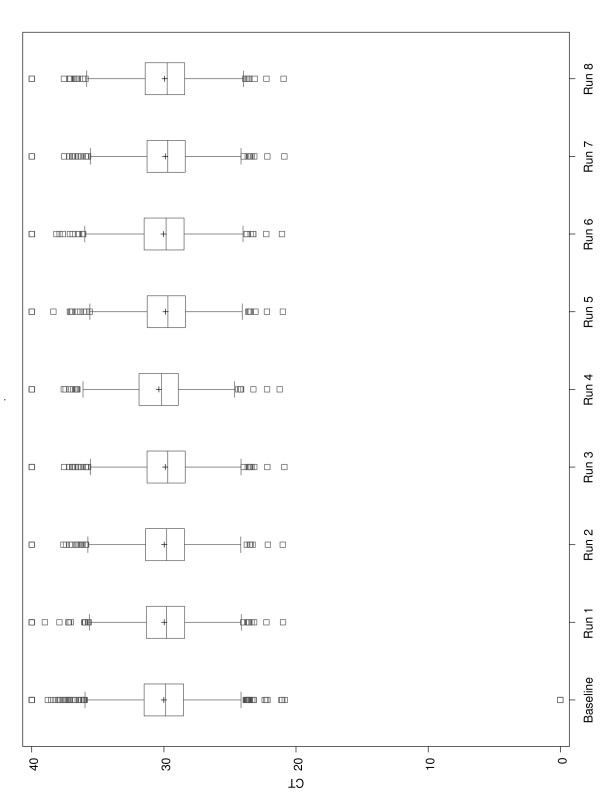
**Boxplots for reference sample CT distributions of the 761 genes at time points throughout the study**. These boxplots show averaged expression values of all 761 genes over the duration of the study. The bottom edge of the box represents the 25^th ^percentile of the data while the top edge of the boxplot represents the 75^th ^percentile. The line inside the box represents the 50^th ^percentile of the data or the median and the symbol + represents the mean. The distance between the 25^th ^and 75^th ^percentiles is defined as the Inter Quartile Range (IQR). A whisker extends from the upper edge of the box to the largest value that is inside a distance of 1.5*IQR. Similarly, a whisker extends from the lower edge of the box to the smallest values inside a distance of 1.5*IQR. Observations outside the fences of 1.5*IQR are marked by a square. The first boxplot represents data from the 3 assay repeats used to set a baseline, all other boxplots represent a single assay run. Overall, the C_T _distributions of the 761 genes assayed in the reference sample were stable throughout the study.

Process reproducibility was monitored throughout the study. Two Tecan Genesis liquid handling robots were used to assemble plates for 335 patient samples over a period of five weeks, for a total of 670 assay plates (this includes samples run with an RNA concentration of less than 1 ng/assay well). Given the large scale of the project and the number of potential sources of variability, we monitored the process performance and reagent stability throughout the duration of the study. This was done by using a single RNA reference sample made by pooling RNA extracted from 80 FPE colon specimens (block age ranging from 3 years – 6 years). As described previously, a pooled FPE sample was selected as a reference RNA since it more representative of a typical study sample than commercially available RNA, yet sufficiently abundant to provide a stable reference baseline throughout the study period. This sample was analyzed 3 times on the same ABI 7900 HT instrument to set a baseline reference profile for the study genes and then was assayed at intervals throughout the duration of the study. Figure 3 shows boxplots of the averaged expression values of all 761 genes over time for the reference sample. The baseline data (3 runs) and all 8 repeat analyses done during the 5 weeks of the study are shown. The outer fence of 3*IQR (Inter Quartile Range) was used to flag outliers. These data demonstrate that assay reagents and the assay processes remained stable throughout the study period. This measurement has been repeated for this sample during subsequent studies and continues to give consistently reproducible results (data not shown).

**Figure 4:**
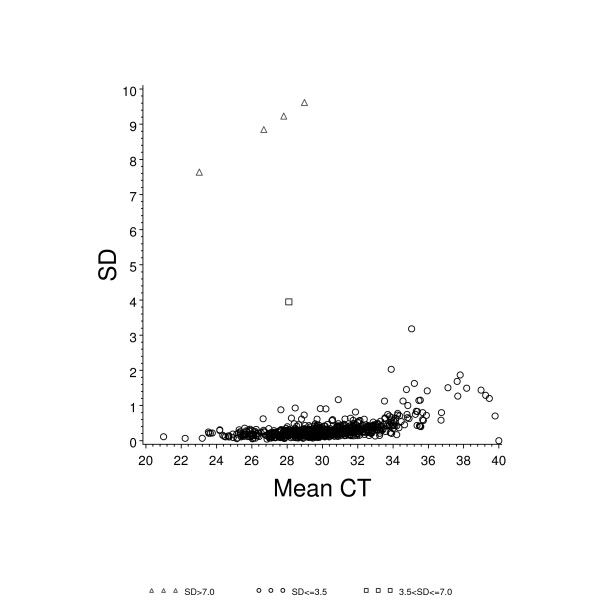
**Average C_T _value versus standard deviation for the reference RNA sample**. Plotted here is the average C_T _value for each gene versus the standard deviation (SD) for each gene from a total of 11 assay runs. The "△" symbols represent genes where a single run had an empty assay well and was assigned a value of zero. The "□" symbol represents a gene where one out of the 11 runs resulted in a spurious "failed" well and was assigned a C_T _value of 40. These data show great consistency over the study.

Figure 4 shows the same reference sample data with the associated standard deviations (SD) obtained for all 11 data points for each gene assay, plotted against the mean C_T _expression value. The majority of assays (737 out of 761) showed a SD less than 1.0. The genes indicated by symbol "Δ"are those where mispipetting by the robot resulted in a single empty well. These wells were given a C_T _value of zero. The gene indicated by symbol "□" was caused by a single spurious failed reaction well which was assigned a C_T _value of 40.

**Figure 5:**
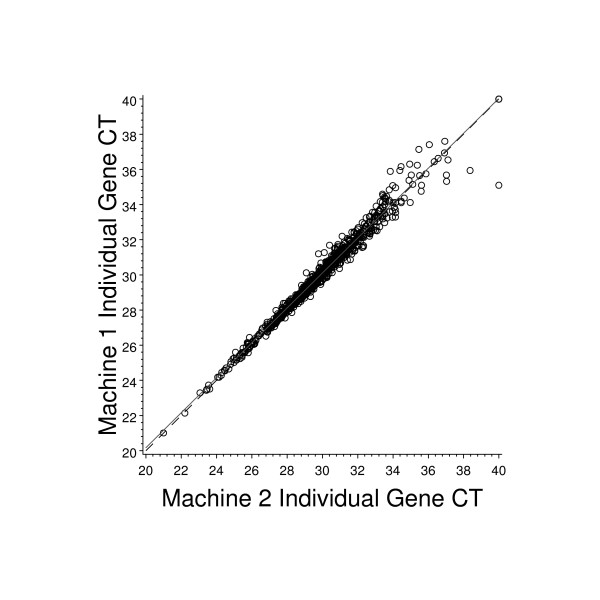
**Comparison of RT-PCR results for the reference RNA sample assayed on two different machines**. Two separate RT-PCR reactions were set up and assayed on different ABI 7900 HT machines, using the reference RNA sample. The graph represents paired raw C_T _values for each of the 761 assays obtained from each of these two machines. The solid line represents the least squares line fit and the dashed line represents the line of concordance.

The four ABI PRISM 7900 HT machines used for the study underwent qualification to meet internal performance specifications prior to use in clinical studies. Figure 5 represents typical data from a comparison (raw C_T _values) of the reference sample on two different machines. The Pearson's correlation is 0.97. The variance in RT-PCR data increases as the C_T _value increases and the assay approaches its "limit of quantitification" (LOQ). This has been described in detail elsewhere [12] and is consistent with the variance observed in this study.

**Figure 6:**
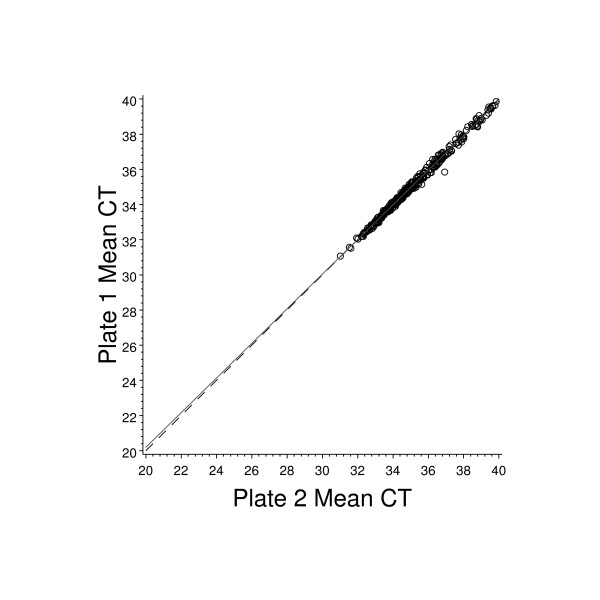
**Comparison of paired whole plate average C_T _values for all patients**. Expression analysis for 761 unique genes required each patient RNA sample to be divided between two 384 well plates. Shown here is the average raw C_T _value for all wells of data obtained for plate one plotted against the average raw C_T _obtained for plate two, for each patient. The patient sample which appears to be an outlier was re-assayed through RT-PCR and on repeat analysis, fell into alignment with the other samples. The solid line represents the least squares line fit and the dashed line represents the line of concordance.

Two 384 well plates were prepared per patient RNA specimen to screen all 761 candidate marker genes. Assays were randomly assigned between the two plates except for reference genes which were present on both assay plates. The average C_T _values for each plate pair were compared and the data are illustrated in Figure 6. This graph demonstrates that plates from the same patient give consistent raw mean C_T _values prior to reference normalization. The one outlier specimen was repeated, and on repeat fell into alignment with the other plate results. It should be noted that Figure 6 represents all patient samples taken through RT-PCR, including the 23 samples run with less than 1 ng/well input RNA. These specimens, which had high average plate C_T _values were excluded from the final study analysis due to unsatisfactory RT-PCR data.

### Expression data normalization

Although the two assay plates for each patient RNA were run on the same ABI Prism 7900 instrument to minimize process variability, data from each sample had to be internally normalized to allow all study specimens to be compared without being confounded by relative variability in RNA quality, quantity or process variability. This was accomplished by subtracting the averaged expression values from 6 reference genes (CLTC, NEDD8, RPLPO, RPS13, UBB, UBC) from the expression values for each gene in each sample. This method has previously been shown to effectively compensate for variability associated with RNA degradation in FPE material of different ages and qualities [5]. Normalization genes were chosen on the basis of low expression variation among patient samples, robust C_T _signals (C_T _< 35) and lack of association with clinical outcome. As part of our quality control and acceptance criteria, the average expression C_T _value for the 6 reference genes for each patient sample had to be less than 35. Within the final evaluable dataset of 270 patient samples, the average reference signal was 29.5 C_T _(SD = 1.39 C_T_) with the lowest observed reference signal at 33.7 C_T_, indicating good overall signal response and relative invariance in reference genes among patients.

**Figure 7:**
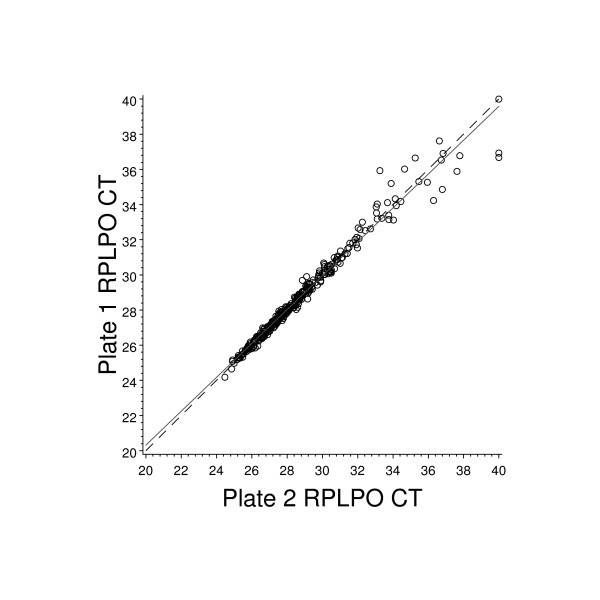
**Comparison of paired raw C_T _values for reference normalization gene RPLPO for all patients**. RPLPO was one of 6 normalization genes. The graph shown here represents the paired raw C_T _values for RPLPO on both assay plates for all patients in the study. Paired plates for each patient sample were assayed on the same ABI 7900 HT machine. The solid line represents the least squares line fit and the dashed line represents the line of concordance.

A high degree of reproducibility was seen for these reference genes, as shown by the RPLPO example, in Figure 7. Because this gene was assayed on both QPCR plates for each patient sample, a direct comparison of C_T _values between plates could be made. The intra-specimen Pearson's correlation for RPLPO was 0.98.

### Gene groups and genes correlated to recurrence free interval (RFI) in colon cancer

**Figure 8:**
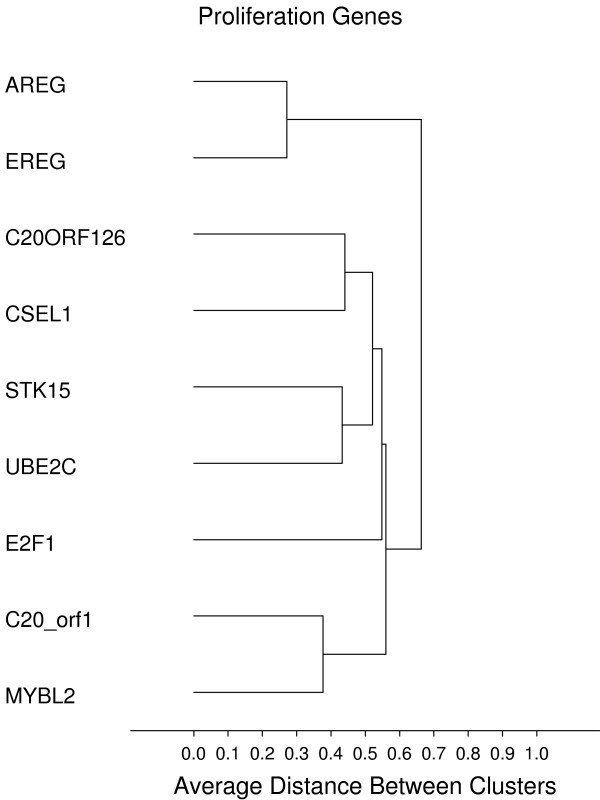
**Proliferation**. **Gene groups identified by clustering analysis**. Clustering analysis was performed using the 1-Pearson's R distance and unweighted pair-group average amalgamation method. Clustering was performed using all 761 genes. Figures 8-13 represent selected clusters from the entire 761 gene dendogram.

**Figure 9:**
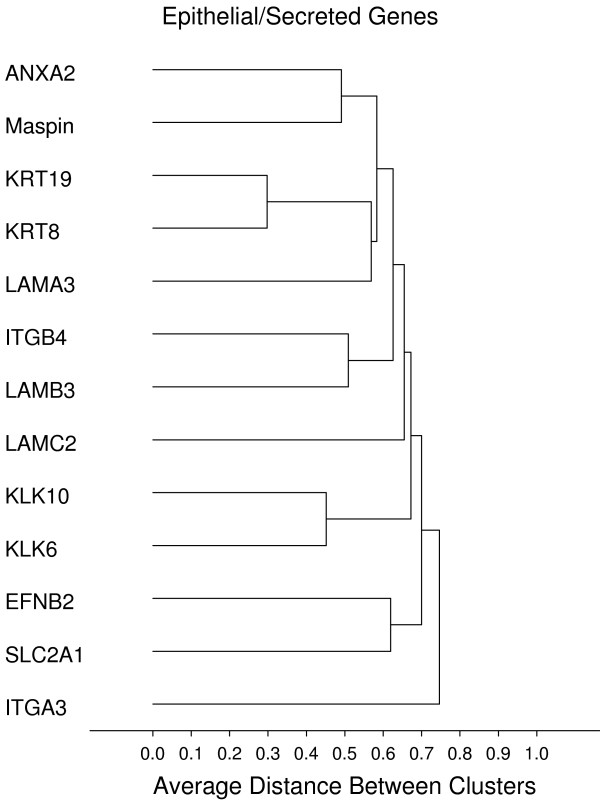
**Epithelial/secreted**. **Gene groups identified by clustering analysis**. Clustering analysis was performed using the 1-Pearson's R distance and unweighted pair-group average amalgamation method. Clustering was performed using all 761 genes. Figures 8-13 represent selected clusters from the entire 761 gene dendogram.

**Figure 10:**
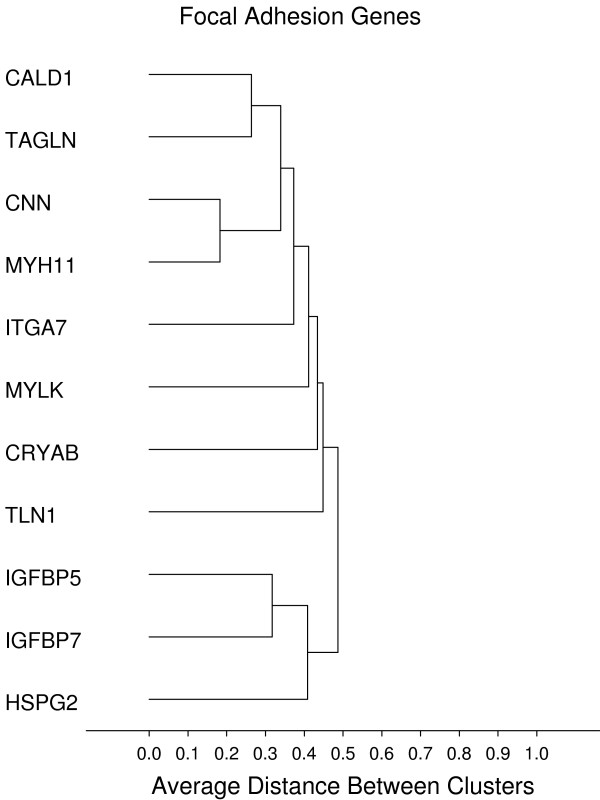
**Focal adhesion**. **Gene groups identified by clustering analysis**. Clustering analysis was performed using the 1-Pearson's R distance and unweighted pair-group average amalgamation method. Clustering was performed using all 761 genes. Figures 8-13 represent selected clusters from the entire 761 gene dendogram.

**Figure 11:**
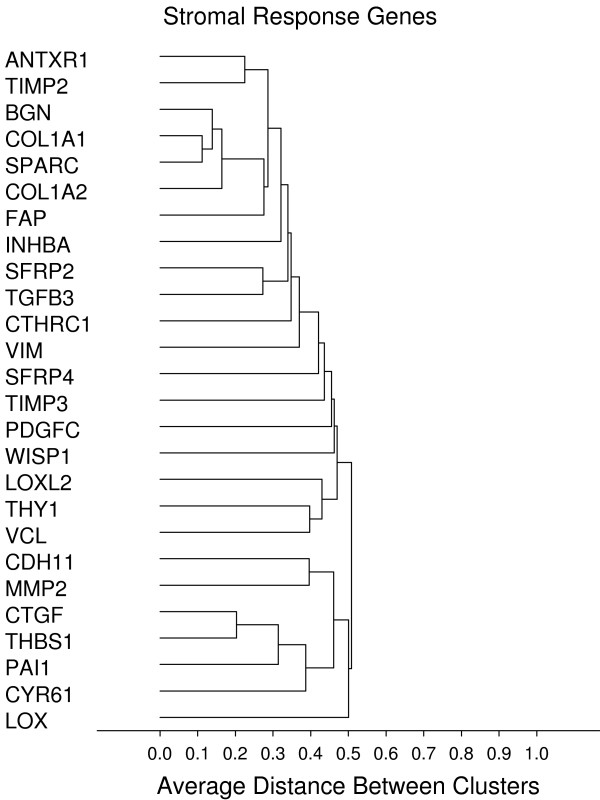
**Stromal response**. **Gene groups identified by clustering analysis**. Clustering analysis was performed using the 1-Pearson's R distance and unweighted pair-group average amalgamation method. Clustering was performed using all 761 genes. Figures 8-13 represent selected clusters from the entire 761 gene dendogram.

**Figure 12:**
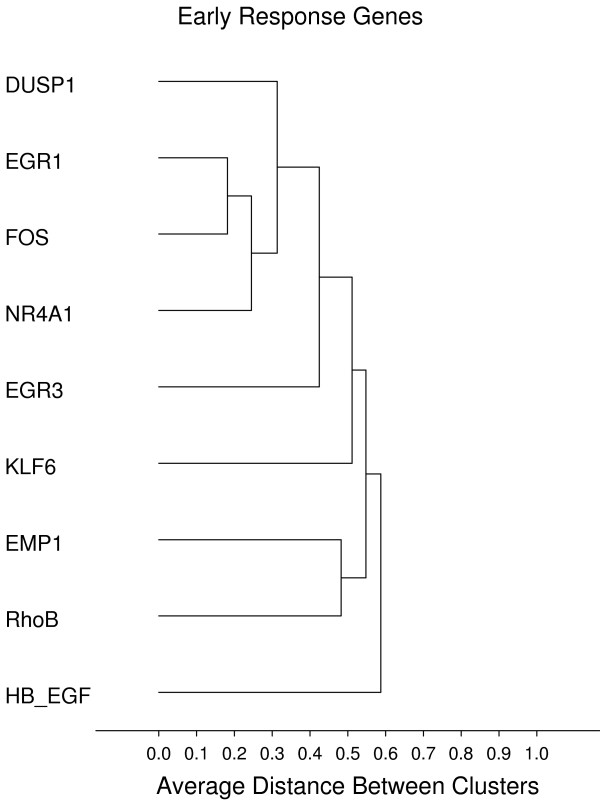
**Early response**. **Gene groups identified by clustering analysis**. Clustering analysis was performed using the 1-Pearson's R distance and unweighted pair-group average amalgamation method. Clustering was performed using all 761 genes. Figures 8-13 represent selected clusters from the entire 761 gene dendogram.

**Figure 13:**
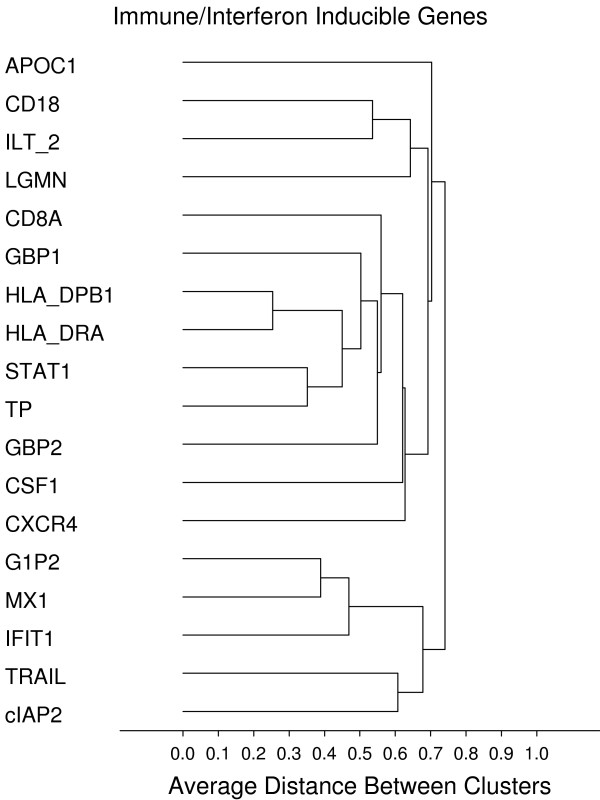
**Immune/interferon-inducible genes**. **Gene groups identified by clustering analysis**. Clustering analysis was performed using the 1-Pearson's R distance and unweighted pair-group average amalgamation method. Clustering was performed using all 761 genes. Figures 8-13 represent selected clusters from the entire 761 gene dendogram.

As an indication of the robustness of this high complexity assay we sought to identify co-expressed groups of genes, that are plausible based on known biological pathways. Unsupervised cluster analysis was performed on the final sample set using all 761 genes, resulting in several distinct clusters representing known biological pathways. Figures 8, 9, 10, 11, 12, 13 display sub-clusters observed among the 761 genes. We identified the following gene groups and pathways: cell cycle and proliferation, epithelial markers (or products secreted by epithelial cells), focal adhesion, stromal response, early response and immune/interferon inducible genes. The most highly correlated gene set from among these groups was the stromal response gene group, where the 1-Pearson's distance for 26 genes was less than 0.5. Within this group are many genes that encode extracellular matrix (ECM) proteins and regulators thereof – a signature similar to that seen during wound healing [15]. There is also a very high concordance in gene expression between p16ink4 and p14arf. This is to be expected since these are alternative transcripts of the same gene and the primer probe set used for p16ink4 also amplifies the p14arf variant. Additionally, correlations among genes within families were observed; for example, CDX1 and CDX2 (Pearsons correlation = 0.67), FUT3 and FUT6 (Pearsons correlation = 0.59), AREG and EREG (Pearsons correlation = 0.73), HSP1A1 and HSP1AB (Pearsons correlation = 0.44).

After data quality control and reference normalization, approximately 19% of the cancer related genes tested in this study were found to have a significant (p < 0.05) correlation with Recurrence Free Interval (RFI) by univariate regression analysis [10]. Approximately one quarter of these genes would be expected to be false positives [10]. While a large number of candidate genes showed significant correlations with clinical outcome (on both the raw and normalized C_T _measurements), the average reference signal was shown not to be correlated with disease recurrence.

## Discussion

The development of a clinically validated test that could determine the risk of recurrence or death from stage II/III colon cancer and the likelihood of benefit from standard chemotherapy regimens is highly desirable but complex. The process begins with biomarker discovery and ends with a clinical validation study with prospectively defined endpoints. Since the method by which an mRNA species is measured will have a profound effect on the success of such a validation study, it is important to characterize and maintain the assay's performance, particularly its reproducibility and quantitative precision. Consequently, we have adopted RT-PCR, the most robust gene expression method available for gene discovery studies. Although nearly 150 genes were found to be significantly related to RFI in this study, some markers will prove to be false positives and true markers will vary in how robustly they correlate with outcome. It is therefore important to evaluate the candidate genes identified here by conducting further independent studies to identify truly useful disease biomarkers. Only after consistent association with clinical outcomes in multiple independent studies should genes be considered for inclusion in an assay used to make clinical decisions. Employing a single technology consistently throughout biomarker discovery and into clinical testing has the advantage of reducing the time required to fully validate and commercialize a multi-gene clinical decision-making tool.

RT-PCR is often carried out using oligo-dT priming to generically reverse transcribe mRNA from the polyA tail. However, this technique is unsuccessful with degraded RNA, such as that extracted from FPE tissue. We have previously shown that RT-PCR using gene-specific priming can be successfully applied to FPE tissue as old as 30 years [5]. As described here, we have now increased the scale of screening using this technique, to 761 genes. We further demonstrate that gene specific priming for 761 assays can be successfully combined into a single RT reaction that results in precise, sensitive and reproducible quantitative PCR for biomarker discovery.

DNA microarrays are a popular technology for biomarker discovery because one can quickly examine the expression of hundreds or thousands of genes. RT-PCR has often been subsequently used to verify the results of microarray data, since it offers much higher sensitivity, specificity, reproducibility and a greater quantitative dynamic range. The present results demonstrate that RT-PCR can also be applied to highly parallel gene expression analysis if robotic processes and assay miniaturization are used. We were able to extract and quantify RNA and generate expression data for 761 unique assays for more than 300 patients in less than 5 weeks.

While screening these patient specimens, it was important to monitor potential sources of variability such as primer and probe stability and gene specific primer pool stability. We used an FPE colon RNA pool as a reference sample and generated a baseline C_T _value for each of the 761 assays. This FPE colon RNA pool reference sample was then included during reverse transcription with every patient sample batch and used to monitor process stability throughout the study. Over the 5 week period, variability within the reference sample remained low, indicating that all patient samples were being analyzed with a stable assay process. Analysis of one of the reference genes, RPLPO, which was assayed on both plates for every patient sample, also highlighted the internal consistency of the process throughout the study.

The robustness of this technology is evidenced by results from hierarchical clustering of all 761 genes which identified known pathways and gene group clusters that one would expect to be co-expressed. One of the largest was a "stromal response" gene group containing genes that are associated with wound healing and are thought to be representative of fibroblast activation, or the 'stromal response' within tumor stroma. Stromal response is becoming increasingly recognized as a marker of invasion and poor clinical outcome in several different classes of solid tumors [16-20]. A number of genes within this group encode proteins that compose or regulate extracellular matrix including BGN, SPARC, CTGF, THBS, VIM, and COL1A1. Several focal adhesion and actin-binding protein genes grouped together to form another distinct cluster, including CALD1, TAGLN, TLN, MYH11, MYLK and CNN. Because MYH11, MYLK and CNN are genes specific to muscle, they could represent a myofibroblast (MF) signature. The myofibroblast cell type has been implicated as a driver of tumor progression [21]. Another cluster might be called an "epithelial/secreted" gene group; within this group are genes known to be markers of epithelial cells or code products secreted by them, such as SERPINB5 (maspin), KRP19, KLK10 and LAMB2. A cluster containing GBP1, GBP2, G1P2, IFIT, CD8A, CD8A, HLA-DRPB1 and CXCR4 represents an immune/interferon-inducible group. Another group contains genes that represent acute response to stimuli, or "early response" genes such as EGR1, EGR3, RhoB, FOS and NR4A. An inflammatory response gene group was also identified, containing genes such as ICAM1, IL1B, IL-8, IL6, OSM and S100A8. A small group of intestinal specific genes such as MUC2, MUC5B, pS2 (TFF1) and TFF3 are also highly correlated in expression. Lastly, it was gratifying to see that the expression of CDH1 and CAPN1 were correlated with one another (Pearson's correlation = 0.48) since CAPN1 cleaves CDH1 [22]. Given the biological connection between these genes, one may have expected this correlation to be higher – possibly indicating specific post-translational control mechanisms may have a role in defining steady state protein levels.

The aim of this study was to identify gene biomarkers that predict recurrence-free interval in patients with Stage II and Stage III colon cancer. Approximately 19% of the 761 genes showed a significant (p < 0.05) association with RFI by univariate Cox proportional hazards regression analysis. It is highly unlikely that any one gene will be able to predict clinical outcome or response to therapy to the extent that it will be useful to oncologists. A successful diagnostic tool is much more likely to consist of a panel of genes and an algorithm weighting and combining each gene contribution into one value that defines the unique risk of recurrence and potential for therapeutic response in each patient. This concept is supported by the observation that several different biological pathways were shown to be associated with RFI in this study.

## Conclusion

We have demonstrated that RT-PCR can be scaled to enable studies testing hundreds of candidate genes for biomarker discovery in hundreds of archival FPE cancer biopsy specimens. Analysis of the data from this study has shown it to be biologically plausible and consistent with known pathways and gene groups identified to be important in cancer. We are applying this technology to colon cancer with the aim of developing a predictive and prognostic clinical test for patients with this disease.

## Methods

### Tissue specimens

Archival colon tumor FPE tissue blocks were provided by the NSABP from both the C01 "A Clinical Trial To Evaluate Postoperative Immunotherapy And Postoperative Systemic Chemotherapy In The Management Of Resectable Colon Cancer" and C-02 "A Protocol To Evaluate The Postoperative Portal Vein Infusion Of 5-Fluorouracil And Heparin In Adenocarcinoma Of The Colon" clinical trials. Patients were enrolled in these trials between 1977–1983. Samples used in this study are representative of the general study populations for both trials.

### Tissue sectioning and macrodissection

Histotechnologists wore gloves at all times when handling tissue blocks. Before and after each block was sectioned, any debris was removed from the microtome with a disposable cotton swab or brush. All appliances (brush, forceps, knife, knife holder base) were wiped with an RNase Zap wipe followed by a soft cloth wetted with de-ionized water. A tissue floatation bath was used to help eliminate wrinkles and distortions in sections being mounted to glass microscope slides. When dissection was required to remove significant non-tumor elements, a representative H&E stained slide was used as a guide to mark tumor and non-tumor portions of three 10 micron unstained slides. Tumor tissue was then scraped away from the non tumor material and placed into an extraction tube. The tumor tissue from all 3 sections was placed into the same tube.

### RNA extraction

RNA was extracted from the tumor-enriched portion of three 10 micron sections per patient block. In order to scale sample throughput to 48 samples/batch, the RNA extraction procedure used a semi-automated method performed on a TECAN robotic liquid handler (TECAN Schweiz AG, Männedorf, Switzerland). The samples originated in individual 1.5 ml Eppendorf tubes and paraffin was removed by incubating with lab grade xylene for 5 minutes. Tissue was then pelleted by centrifugation at room temperature (+18°C to +25°C) for 5 minutes at approximately 14,000 RPM. The xylene was removed and the procedure repeated. The tissue pellet was washed by inverting several times with 200 proof ethyl alcohol and again pelleting by centrifugation at room temperature. The ethyl alcohol wash step was repeated. Immediately prior to adding Proteinase K, samples were inspected for residual alcohol; if any alcohol was visible, it was aspirated without disturbing the tissue pellet. Proteinase K digestion was performed using reagents from the MasterPure^® ^Purification kit (Epicentre, Madison, WI). Samples were incubated at +65°C for 2 hr with Proteinase K. Protein and genomic DNA were removed by manual addition of an equal volume of acid-phenol: chloroform and the removal of the upper aqueous phase after centrifugation for 5 minutes at approximately 10,000 RPM. Samples tubes were transferred to a TECAN liquid handler where purification was completed using the mirVana™ RNA purification kit (Ambion, Austin, Texas) on a 96 well glass fiber filter plate. Purification was followed by DNase I treatment on the same 96 well filter plate.

### RNA quantitation

RNA was quantified using the RiboGreen fluorescence method as described by the kit manufacturer (Invitrogen, Carlsbad, CA.).

### Candidate gene selection

Candidate genes were obtained from published gene expression profiling data relating to colon cancer prognosis and response to therapy, biological pathways known to be important in cancer [23-28] and suggestions by our collaborators at the NSABP. The full gene list, accession numbers and associated primer and probe oligo sequences are provided [see Additional file 3].

### TaqMan^® ^primer and probe design

The reference sequence for each gene included in the study was obtained from the NCBI Entrez website. TaqMan^® ^RT-PCR primers and probes were designed using an automated in-house primer design module. The complete list of assay primers and probes is shown in Additional file 3. Oligonucleotides were purchased from Biosearch Technologies Inc. (Novato, CA), Integrated DNA Technologies (Coralville, IA) and Eurogentec (San Diego, CA). Dual labeled TaqMan^® ^probes had 5'FAM as a reporter and either 3'BHQ-1 or 3'BHQ-2 as a quencher. Amplicon size was limited to a maximum of 90 bp.

### Reverse transcription

Reverse transcription was performed using the Omniscript kit (Valencia, CA) for RT-PCR. For initial investigations, reverse primers (each at 100 μM) were collected into sub-pools of 94–96 primers. An aliquot from each sub-pool was added together to create the final GSP pool (each primer at 100 nmol/L). For the clinical study, reverse primers (each at 1 mM) were first collected in sub-pools of 89–96 primers and tested for priming performance before being combined into a master 761 gene-specific primer pool (each primer at 1 μmol/L). For both the initial investigations and the clinical study, each primer in the RT reaction was at a concentration of 50 nmol. Our standard procedure was to add RNA to the RT reaction at 12.5 ng/ul which equates to 1 ng/well (cDNA) for quantitative PCR. In each case, the RT reaction was performed in a single tube with the GSP pool (ie up to 761 reverse primers). The resulting cDNA was distributed equally among the wells of a 384 well plate, and the appropriate forward and reverse primer and probe were added to each assay well.

### TaqMan^® ^gene expression analysis

For each patient sample, two 384-well plates were used. Assays for 7 potential reference genes were included on both plates, with all other gene assays randomly distributed in single assay wells. RT-PCR assays for three K-ras gene mutations and one BRAF gene mutation were included in the assay panel along with each corresponding wild type allele assay. Therefore, 757 normal gene alleles were assayed and 4 mutant genes were assayed, bringing the total number of unique assays to 761. TaqMan^® ^RT-PCR was performed according to instructions of the manufacturer, using Applied Biosystems Prism (ABI) 7900 HT instruments. Reactions were performed in a 5 μl volume with cDNA equivalent to 1 ng total RNA. Final primer and probe concentrations were 0.9 μmol/L (primers) and 0.2 μmol/L (probe). For the K-ras mutation assays a blocker oligomer was added to the primer and probe pool at a final concentration of 3.6 μmol/L (mutant 1) 3.6 μmol/L (mutant 2) and 12.95 μmol/L (mutant 3). These blockers are added to inhibit amplification of the non-mutant allele, permitting specific amplification of the mutant allele. PCR cycling conditions were 95°C for 10 minutes for one cycle, 95°C for 20 seconds, and 60°C for 45 seconds for 40 cycles. A reference sample (pooled colon FPET RNA) was assayed throughout the study to ensure reagent and process stability. As a negative control, wells without any template were also assayed every two weeks to ensure that no exogenous nucleic acid contaminations occurred.

### Unsupervised hierarchical clustering

The unsupervised hierarchical clustering of genes was performed using 1-Pearson R as the distance measure for gene expression and the un-weighted pair-group average as the amalgamation method [29].

## Authors' contributions

KMCL lead the study and provided scientific input for the manuscript. JYW and AC prepared reagents, extracted and processed the samples. CS, JRH and GY provided biostatistical input. JLS and AN lead the development of the robotic systems. JB provided scientific input for the manuscript. CK reviewed blocks and slides from NSABP colon protocols and selected the appropriate blocks for processing, and MTC provided scientific input for the manuscript and managed the project.

GY and CK have no competing interests to declare. KMCL, JYW, AC, CS, JRH, JLS, AN, JB and MTC were all full-time employees of Genomic Health at the time this work was done.

## Supplementary Material

Additional file 1 **Random hexamer versus gene specific priming using highly degraded RNA as a template**. The 21 genes from the OncotypeDX Breast Cancer assay were assayed using highly fragmented RNA as template. Random hexamer priming (RH) or gene specific pool (GSP) priming in the reverse transcription reaction were compared. Each gene was run in triplicate wells and the data shown represents the average and standard deviation. The increased specificity of GSP priming allows even the most highly degraded sample to be amplified with robust signal. Click here for file

Additional file 2 **Random hexamer versus gene specific priming using in tact RNA as a template**. The 21 genes from the OncotypeDX Breast Cancer assay were assayed using Commercially available, in tact RNA as template. Random hexamer priming (RH) or gene specific pool (GSP) priming in the reverse transcription reaction were compared. Each gene was run in triplicate wells and the data shown represents the average and standard deviation. There is only a modest benefit from using GSP priming versus random priming when the template is of high quality. Click here for file

Additional file 3 **Gene list and associated primer and probe oligo sequences**. Listed are the Official Symbols of all genes analyzed in the study. The forward, reverse and probe oligo sequences are also shown. Probes were dual labelled 5'FAM as a reporter and either 3'BHQ-1 or 3'BHQ-2 as a quencher. Click here for file
