# Supplementary material for: Biomarker discovery for colon cancer using a 761 gene RT-PCR assay
Source: BMC Genomics. 2007 Aug 15;8:279. doi: 10.1186/1471-2164-8-279 (PMC1994687; doi:10.1186/1471-2164-8-279)
Supplement: Supplementary file 1 [file 1471-2164-8-279-S1.doc]

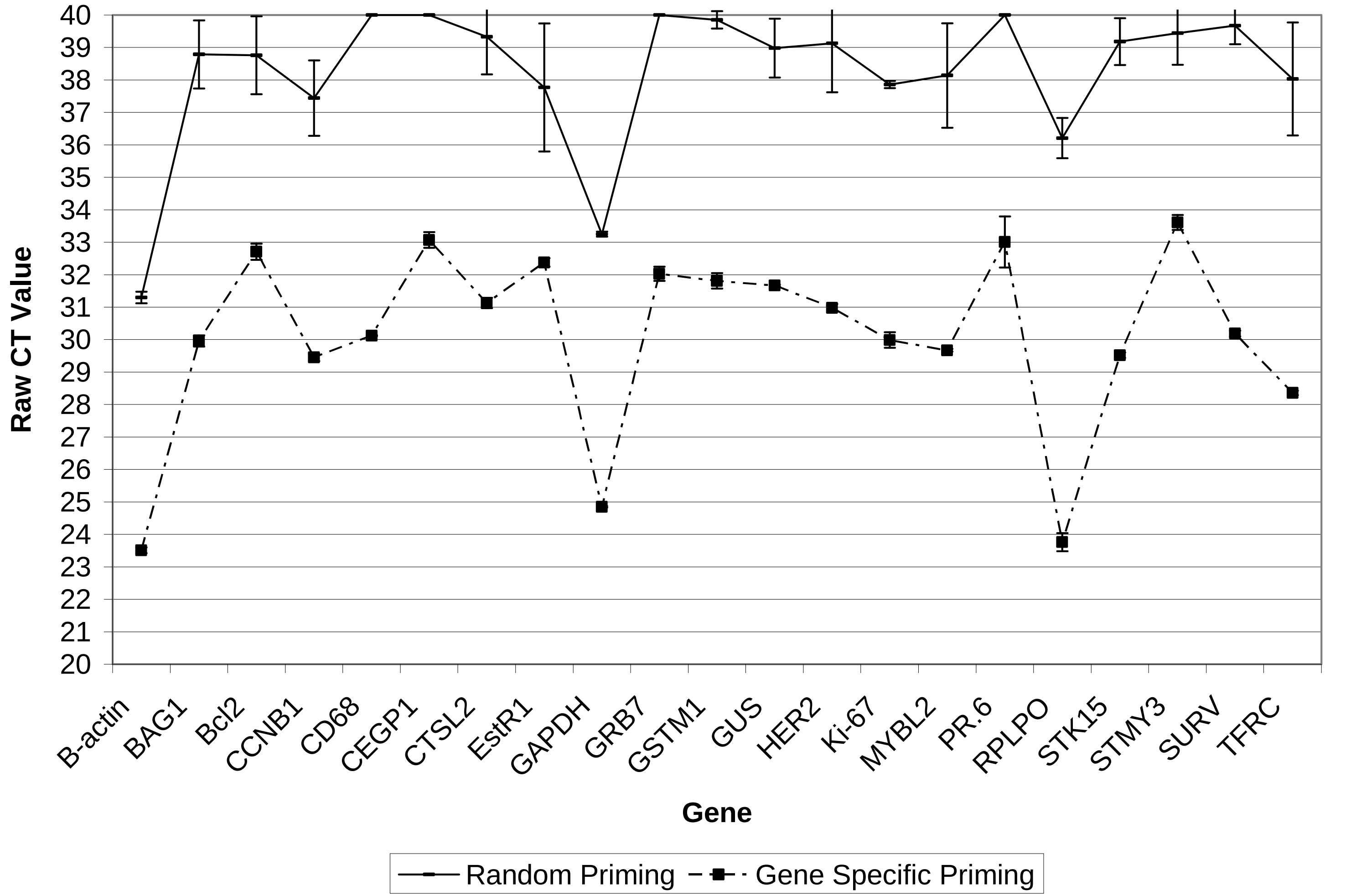

Raw CT Value
40
39
38
37
36
35
34
33
32
31
30
29
28
27
26
25
24
23
22
21
20
B-actin
BAG1
Bcl2
CCNB1
CD68
CEGP1
CTSL2
EstR1
GAPDH
GRB7
GSTM1
GUS
HER2
Ki-67
MYBL2
PR.6
RPLPO
STK15
STMY3
SURV
TFRC
Gene
Random Priming
Gene Specific Priming
